# A study of the endohelminths of the European perch *Perca fluviatilis* L. from the central region of the Danube river basin in Slovakia

**DOI:** 10.3897/zookeys.899.39638

**Published:** 2019-12-12

**Authors:** Ľudmila Juhásová, Alžbeta Radačovská, Eva Bazsalovicsová, Dana Miklisová, Marcela Bindzárová-Gereľová, Ivica Králová-Hromadová

**Affiliations:** 1 Institute of Parasitology, Slovak Academy of Sciences, Hlinkova 3, 040 01 Košice, Slovakia Institute of Parasitology, Slovak Academy of Sciences Košice Slovakia; 2 Institute of Geodesy, Cartography and Geographical Information Systems, Technical University of Košice, Faculty of Mining, Ecology, Process Control and Geotechnologies, PK 19, 04120 Košice, Slovakia Technical University of Košice Košice Slovakia

**Keywords:** endoparasites, *Clinostomum complanatum*, *Eustrongylides* sp., Percidae, *Proteocephalus percae*, *Triaenophorus nodulosus*

## Abstract

The European perch *Perca fluviatilis* L. serves as a host of different endohelminths of Trematoda, Cestoda, Nematoda, and Acanthocephala. Its natural range covers freshwater basins throughout much of Europe, including the Danube. Since information about endohelminths of European perch from this international river basin has been rather sporadic, the parasitological examinations of 700 perch from the central region of the Danube river basin in Slovakia were performed in October 2017 and April 2018. The larval stages of *Triaenophorus nodulosus* (Cestoda) were found in cysts located in the perch liver and adults of *Proteocephalus percae* (Cestoda) were isolated from the intestine. The larval stages of *Eustrongylides* sp. (Nematoda) and metacercariae of *Clinostomum complanatum* (Trematoda), both potential causative agents of fish-borne zoonoses, were found in the musculature. Spatial and seasonal differences in the occurrence of currently detected helminths were discussed with data on biological and environmental conditions of particular sampling site.

## Introduction

The Danube is the second longest river in Europe shared by 10 European countries, including Germany, Austria, Slovakia, Hungary, Croatia, Serbia, Romania, Bulgaria, Moldova, and Ukraine. The Danube river basin, one of the most international river basins in the world (Liska 2015), is divided into Upper, Middle, and Lower basins (Liška et al. 2008). The largest part is the Middle Basin, which includes the area from Bratislava in Slovakia to the Iron Gate dams at the border of Serbia and Romania (Paunovic et al. 2007).

The Danube represents an important ecosystem with a high biodiversity of aquatic organisms (Tockner et al. 1998). A study on the fish fauna of the entire course of the Danube revealed the presence a high diversity of some 100 fish species, including cyprinids, silurids, esocids, percids, anguillids, and salmonids (Schiemer et al. 2004). Due to its international character and rich fish fauna, the Danube also plays a notable role in the spreading of various parasitic and infectious fish diseases.

Percids represent a so-called promising fish species for a fishery and aquaculture (Kestemont et al. 2015). The European perch, *Perca fluviatilis* Linnaeus, 1758, is an ecologically significant predator and popular sport fish noted for its fighting qualities and taste (Popova and Sytina 1977). It is among the most common and widely distributed members of the Percidae throughout Europe (Giannetto et al. 2012), including the Danube.

The European perch serves as a host for different endohelminths (Trematoda, Cestoda, Nematoda, and Acanthocephala). However, only a few parasitological studies have been conducted on the European perch from the Danube since the 1980s. All of them were in the Lower Basin, in particular in Srebarna Lake (north-eastern Bulgaria), which is connected via an artificial canal to the Bulgarian part of the Danube (Kakacheva-Avramova 1983; Shukerova et al. 2010; Atanasov 2012; Kirin et al. 2013). Since no recent information about endohelminths of the European perch from the Middle Danube is available, parasitological examinations of perch from five selected localities in the Slovak part of the river were performed in two periods of the year. The spatial and seasonal differences in the occurrence of detected endohelminths were discussed with data on biological and environmental conditions of particular sampling site.

## Material and methods

### Material

**Figure 1.**
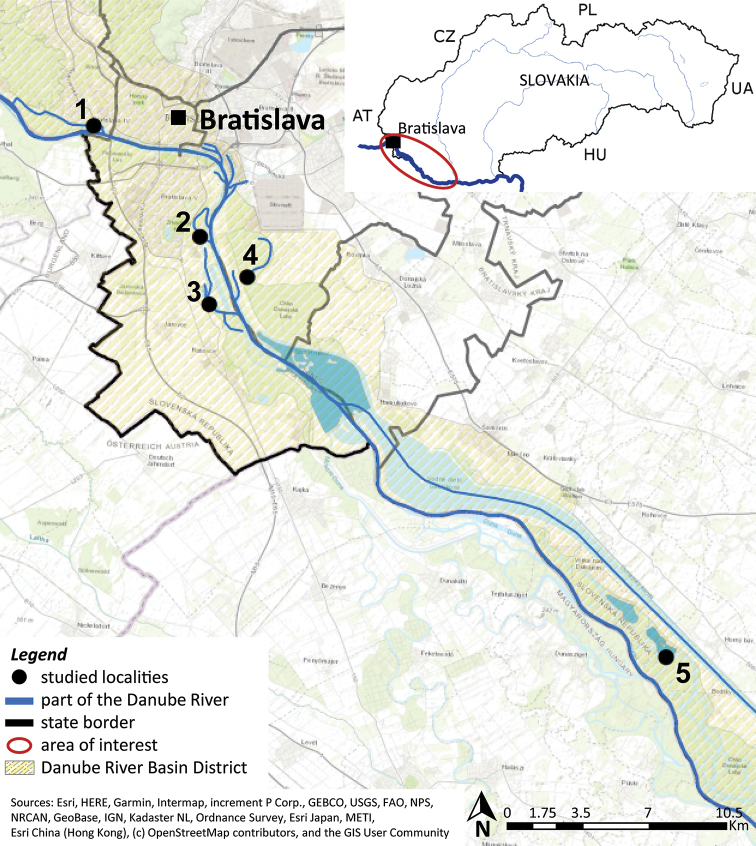
The schematic presentation of sampling sites in Slovak part of the Danube. 1, Karloveské river branch (48°8'46.08"N, 17°3'50.33"E); 2, Starohájske river branch (48°6'11.50"N, 17°7'56.19"E); 3, Jarovecké river branch (48°4'32.34"N, 17°8'23.90"E); 4, Biskupické river branch (48°5'15.45"N, 17°9'44.21"E); 5, Šulianske Lake (47°56'26.66"N, 17°25'42.55"E).

The European perch were collected from the central region of the Danube, in particular from four river branches (RB) located next to the main stream and from Šulianske Lake, a gravel pit permanently flooded with water and near the Danube (Fig. 1).

In total, 700 individuals of European perch (length 107–165 mm; 71.4% females and 28.6% males) from all localities were caught by professional fishermen in October 2017 and April 2018. The number of fish obtained during both seasons was approximately equal (October 49.4%; April 50.6%; for more details see Table 2). Incomplete parasitological necropsy included a detailed examination of the peritoneal cavity, intestine, liver and other abdominal organs. In order to examine the musculature of perch, thin (approximately 5 mm) slices of fillets of whole fish were examined. The skin, gills, and eyes were not investigated. The parasites were washed in a physiological solution and observed under a stereoscopic microscope for morphological identification to genus and/or species level using taxonomic keys (Moravec 1994; Scholz and Hanzelová 1998; Gibson et al. 2002; Kuchta et al. 2008).

### Molecular genotyping

The parasites were rinsed in physiological solution and fixed in 96% ethanol immediately after dissection. Taxonomic identification of the parasites to the species level was performed by molecular genotyping using a partial small subunit of the nuclear ribosomal RNA gene (ssrDNA) as a molecular marker. For PCR amplification and sequencing of ssrDNA, the following universal primers were applied: WormA (5'–GCGAATGGCTCATTAAATCAG–3') and WormB (5'–CTTGTTACGACTTTTACTTCC–3') (Littlewood and Olson 2001). Details on PCR amplification, sequencing and sequence analysis were published in Bazsalovicsová et al. (2018). The data obtained were compared with sequences deposited in the GenBank database (https://www.ncbi.nlm.nih.gov/).

### Statistical tests

Fisher’s exact test was used to compare the prevalence of endohelminths from the five studied localities between the two seasons. The samples were initially analysed as separate observations of the locality per season for each parasite species, then consequently evaluated independently to the examination timing for each locality. A *p* value under 0.05 was considered to be significant. Moreover, 95% confidence intervals (CI) were calculated individually for each proportion. The statistical analyses were performed by the Quantitative Parasitology on the Web (Reiczigel et al. 2019).

## Results

Of the 700 European perch examined from five sampling sites in the Middle Danube river basin in Slovakia in October 2017 and April 2018, 176 were found to be infected (prevalence 25.1%; CI 22.0–28.5%). Endohelminths were determined in all the studied localities; however, species composition and prevalence varied between different sampling sites and/or examination timing.

Two tapeworms were found in European perch; larval stages of *Triaenophorus nodulosus* (Pallas, 1781) Rudolphi, 1793 (Bothriocephalidea) were found in cysts localized in the liver, and juveniles to adults (at different stages of maturity) of *Proteocephalus percae* (Müller, 1780) (Proteocephalidea) were isolated from the pyloric caeca. In the musculature, the larval stages of the nematodes of the genus *Eustrongylides* Jägerskiöld, 1909 (Dioctophymatoidea) and metacercariae of the fluke *Clinostomum complanatum* (Rudolphi, 1814) Braun, 1899 (Diplostomida) were detected.

To confirm the taxonomic status of all detected species, ssrDNA was applied as the molecular marker for genotyping. After PCR amplification, a 730 bp fragment was obtained, sequenced and compared with sequences of respective species deposited in the GenBank. The ssrDNA sequence of *T. nodulosus* from our study was 100% identical with *T. nodulosus* from pike (*Esox lucius*) from Scotland (GenBank Accession number KR780923; Brabec et al. 2015), and the one of *P. percae* was 100% identical with *P. percae* from European perch from Switzerland (KX768934; Scholz et al. 2017). The nematode *C. complanatum* corresponded (100% identity) with *C. complanatum* from Italy (FJ609420; Gustinelli et al. 2010). The sequence of *Eustrongylides* sp. was 99.1% identical with *Eustrongylides* sp. from dwarf snakehead (*Channa gachua*) from India (MG696298; unpublished).

**Note**: Although more than 20 species of the genus *Eustrongylides* were originally described, the validity of many of them is disputable (Eberhard and Ruiz-Tiben 2014). A revision of the genus revealed that there are three valid species: the type species *Eustrongylides tubifex* (Nitzsch & Rudolphi, 1819) Jägerskiöld, 1909; *Eustrongylides ignotus* Jägerskiöld, 1909; and *Eustrongylides excisus* Jägerskiöld, 1909 (Measures 1988). Although *E. excisus* and *E. tubifex* have previously been reported from perch in the Lower Danube (Table 1), there are no data on any DNA region of both nematodes available in the GenBank database. The sequences obtained in the current study corresponded to the sequence data on species assigned as *Eustrongylides* sp.

**Table 1.** Summary of the literature data (1980–2019) of endohelminths detected in European perch *Perca fluviatilis* L. in the Danube.

Parasite	Locality	Season	No.	P (%)	Dev. stage	References
** CESTODA **						
*Proteocephalus percae*	Srebarna Lake, NE Bulgaria	autumn	60	3.3	A	Shukerova et al. 2010
		spring	60	1.7	A	Shukerova et al. 2010
** TREMATODA **						
*Bolboforus confusus*	Srebarna Lake, NE Bulgaria	spring	60	3.3	M	Shukerova et al. 2010
		summer	60	10.0	M	Shukerova et al. 2010
		autumn	60	1.7	M	Shukerova et al. 2010
*Diplostomum pseudospathaceum*	Srebarna Lake, NE Bulgaria	autumn	60	1.1	M	Shukerova et al. 2010
	River Danube, Bulgaria	n.a.	40	20.0	M	Atanasov 2012
*Diplostomum spathaceum*	Srebarna Lake, NE Bulgaria	spring	60	3.3	M	Shukerova et al. 2010
		summer	60	1.7	M	Shukerova et al. 2010
*Ichthyocotylurus pileatus*	Srebarna Lake, NE Bulgaria	summer	60	3.3	M	Shukerova et al. 2010
*Posthodiplostomum cuticola*	Srebarna Lake, NE Bulgaria	summer	60	1.7	M	Shukerova et al. 2010
*Tylodelphys clavata*	Srebarna Lake, NE Bulgaria	spring	60	56.7	M	Shukerova et al. 2010
		summer	60	81.7	M	Shukerova et al. 2010
		autumn	60	86.7	M	Shukerova et al. 2010
** NEMATODA **						
*Contracaecum microcephalum*	Srebarna Lake, NE Bulgaria	autumn	60	3.3	L	Shukerova et al. 2010
*Eustrongylides excisus*	Srebarna Lake, NE Bulgaria	spring	60	8.3	L	Shukerova et al. 2010
		summer	60	10.0	L	Shukerova et al. 2010
		autumn	60	23.3	L	Shukerova et al. 2010
	River Danube, Bulgaria	n.a.	40	7.5	L	Atanasov 2012
	Srebarna Lake, NE Bulgaria	summer	n.a.	100	L	Kirin et al. 2013
	River Danube, Bulgaria	summer	n.a.	100	L	Kirin et al. 2013
*Eustrongylides tubifex*	Srebarna Lake, NE Bulgaria	autumn	60	1.7	L	Shukerova et al. 2010
*Rhaphidascaris acus*	Srebarna Lake, NE Bulgaria	summer	60	10.0	L	Shukerova et al. 2010
** ACANTHOCEPHALA **						
*Acanthocephalus lucii*	River Danube, Bulgaria	n.a.	n.a.	n.a.	n.a.	Kakacheva-Avramova 1983
	Srebarna Lake, NE Bulgaria	spring	60	1.7	A	Shukerova et al. 2010
*Acanthocephalus anguillae*	Srebarna Lake, NE Bulgaria	spring	60	1.7	A	Shukerova et al. 2010

No. number of fish examined, P prevalence, Dev. stage developmental stage, NE north-eastern, n.a. not available, A adults, L larvae, M metacercariae.

### *Triaenophorus nodulosus* (larvae)

**Figure 2.**
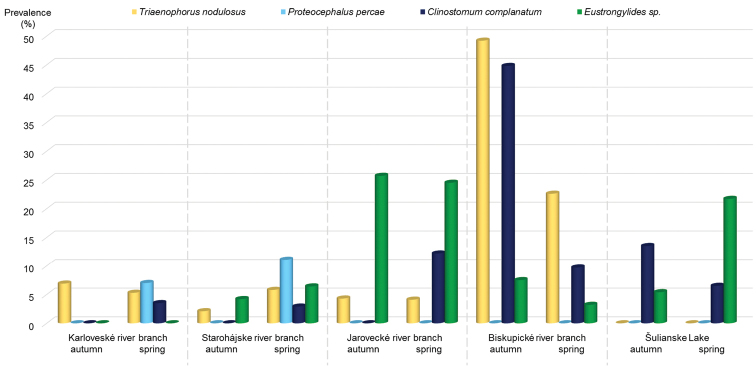
Schematic presentation of prevalence of the parasites found in the five studied localities in autumn and spring.

The mean intensity of infection (MI) for *T. nodulosus* from all five localities ranged between 1.0 and 9.5 (Table 2). The overall highest prevalence was observed for *T. nodulosus* in perch from the Biskupické RB, with higher values in October (P = 49%) than in April (P = 23%). On the contrary, larvae of this tapeworm were not detected in Šulianske Lake irrespective of the season (Fig. 2; Table 2). There were no statistically significant differences between sampling periods in all studied localities, except for the Biskupické RB (*p* < 0.05) (Table 2).

### *Proteocephalus percae* (adult)

The MI for this intestinal tapeworm was 2.3 and 4.0 (Table 2). It was detected in two out of five localities, in particular in the Karloveské RB (P = 7.0%) and Starohájske RB (P = 11.1%); at both localities *P. percae* was present only in spring (Fig. 2, Table 2).

**Table 2.** Statistical data on detected endohelminths of European perch *Perca fluviatilis* L. from studied localities in the Danube river basin, Slovakia.

**Locality**	**TE**	**No.**	***Triaenophorus nodulosus* (Cestoda)**					***Proteocephalus percae* (Cestoda)**				
			**IF**	**MI (max)**	**P (%)**	**FET**	**95% CI**	**IF**	**MI (max)**	**P (%)**	**FET**	**95% CI**
Karloveské RB	Oct/17	29	2	9.5 (15)	7	ns	1–23	0	–	–	–	–
	Apr/18	57	3	1.0 (1)	5	ns	1–15	4	4.0 (4)	7	ns	2–17
Starohájske RB	Oct/17	143	3	3.0 (4)	2.1	ns	0.4–6.0	0	–	–	–	–
	Apr/18	171	10	2.8 (12)	5.8	ns	2.8–10.5	19	2.3 (8)	11.1	***	6.8–16.8
Jarovecké RB	Oct/17	70	3	1.3 (2)	4	ns	1–12	0	–	–	–	–
	Apr/18	49	2	5.0 (9)	4	ns	5–14	0	–	–	–	–
Biskupické RB	Oct/17	67	33	7.1 (20)	49	*	37–62	0	–	–	–	–
	Apr/18	31	7	6.4 (12)	23	*	10–41	0	–	–	–	–
Šulianske Lake	Oct/17	37	0	–	–	–	–	0	–	–	–	–
	Apr/18	46	0	–	–	–	–	0	–	–	–	–
**In total**		**700**	**63**	**5.6 (20)**	**9.0**	–	**7.0–11.4**	**23**	**2.3 (8)**	**3.3**	–	**2.1–4.9**
			***Clinostomum complanatum* (Trematoda)**					***Eustrongylides* sp. (Nematoda)**				
			**IF**	**MI (max)**	**P (%)**	**FET**	**95% CI**	**IF**	**MI (max)**	**P (%)**	**FET**	**95% CI**
Karloveské RB	Oct/17	29	0	–	–	–	–	0	–	–	–	–
	Apr/18	57	2	2.0 (2)	4	ns	1–12	0	–	–	–	–
Starohájske RB	Oct/17	143	0	–	–	–	–	6	1.3 (2)	4.2	ns	1.6–8.9
	Apr/18	171	5	1.0 (1)	2.9	ns	1.0–6.7	11	1.9 (10)	6.4	ns	3.3–11.2
Jarovecké RB	Oct/17	70	0	–	–	–	–	18	1.4 (5)	26	ns	16–38
	Apr/18	49	6	2.0 (6)	12	**	5–25	12	2.2 (7)	24	ns	13–39
Biskupické RB	Oct/17	67	30	3.4 (23)	45	***	33–57	5	1.0 (1)	8	ns	2–17
	Apr/18	31	3	2.0 (2)	10	***	2–26	1	1.0 (1)	3	ns	0–17
Šulianske Lake	Oct/17	37	5	1.6 (4)	14	ns	4–28	2	1.0 (1)	5	ns	7–18
	Apr/18	46	3	1.0 (1)	6	ns	1–18	10	2.8 (7)	22	ns	11–36
**In total**		**700**	**54**	**2.6 (23)**	**7.7**	–	**5.8–9.9**	**65**	**1.8 (10)**	**9.3**	–	**7.2–11.7**

RB river branch, TE timing of examination, Oct/17 October 2017, Apr/18 April 2018, No. number of fish examined, IF number of infected fish, MI mean intensity of infection, max maximum number of parasites, P prevalence, FET Fisher’s exact test of seasonal differences in prevalence for each locality separately, 95% CI confidence interval, ns nonsignificant, * *p* < 0.05, ** *p* < 0.01, *** *p* < 0.001

### *Clinostomum complanatum* (metacercariae)

The mean intensity of infection ranged between 1.0–3.4 (Table 2). The highest prevalence (45%) was detected in the Biskupické RB in October and markedly lower values (P = 10%) were recorded in April (Fig. 2; Table 2). A similar seasonal pattern was observed in Šulianske Lake (October, P = 14%; April, P = 6%). While there was high statistical support for the results in the Biskupické RB (*p* < 0.001), data detected in Šulianske Lake were statistically nonsignificant (Table 2). Opposite results were observed in the three remaining localities, where metacercariae of *C. complanatum* were not detected in October but were present in April (Fig. 2; Table 2).

### *Eustrongylides* sp. (larvae)

The MI values ranged between 1.0–2.8 (Table 2). The larvae of *Eustrongylides* sp. were detected in perch musculature at the highest prevalence in the Jarovecké RB, where no striking differences were detected between October (P = 26%) and April (P = 24%) (Fig. 2; Table 2). A similar prevalence (P = 22%) was detected in Šulianske Lake in spring, while lower prevalence (P = 5%) was recorded in October (Fig. 2; Table 2). Larvae of *Eustrongylides* sp. were not detected in the Karloveské RB (Fig. 2; Table 2).

## Discussion

Over the last four decades, several species of flukes and nematodes, two species of thorny-headed worms and single tapeworm have been found in European perch from the Danube (for details, see Table 1 and references therein). In the current study, only four endohelmints were detected in perch from the central region of the river. Metacercariae of *C. complanatum* were detected for the first time, while *P. percae* and *Eustrongylides* sp. were previously found in Srebarna Lake in north-eastern Bulgaria (Shukerova et al. 2010; Kirin et al. 2013). The only record of the presence of *T. nodulosus* in perch from the Danube was published more than 60 years ago (Dyk 1955). Spatial and seasonal differences in the occurrence of currently detected helminths could be explained by diverse environmental conditions of particular sampling site and by an availability of suitable definitive hosts.

The occurrence of *T. nodulosus* in the studied localities was rather diverse; it was absent in Šulianske Lake, while low values of prevalence were documented in Karloveské, Starohájske and Jarovecké RB. The highest prevalence was detected in the Biskupické RB, a branch of the river about 20 m wide and connected to the Danube by an artificial channel (Jursa 2003). Water in the stream branch has rich fish diversity, and it is regularly restocked with various fish species, including perch (second intermediate host) and pike (definitive host of *T. nodulosus*). The high prevalence of *T. nodulosus* in this particular RB may be related to the fact that 5000 individuals of pike were restocked in the Biskupické RB in December 2015 (http://cokdezakolko.sk/category/zarybnenie/; in Slovak).

The prevalence of *T. nodulosus* in the Biskupické RB was significantly higher in autumn. On the contrary, no evident seasonal variation was detected in three other studied localities. Since plerocercoids can live in the intermediate fish host up to three years, little or no seasonal variations have been previously detected in periodicity of *T. nodulosus* in perch. Besides, the dynamics of infections and maintenance of plerocercoids in fish may vary considerably from water to water (Chubb 1980 and references therein).

The second tapeworm detected in the current work, *P. percae*, was present in Karloveské and Starohájske RB only in spring (April). Similar seasonal dynamics with the maximum values of prevalence in March and April were also observed by Scholz (1986) and Chubb (1982), respectively.

The two remaining helminths, *C. complanatum* and *Eustrongylides* sp., utilize birds as definitive hosts. The Protected Bird Area of the Danube floodplain is a refuge for tens of thousands of birds; it is an internationally important breeding area, nesting site, migration corridor, and wintering place of migratory and resident birds, such as mallard, great crested grebe, cormorant, black stork and other long-necked wading birds, which serve as definitive host of the above species. This has evidently played an important role in a broad spatial distribution of both endohelminths; while *C. complanatum* was detected in all five studied localities, *Eustrongylides* sp. was absent only in Karloveské RB.

Whereas birds are preferable hosts of *C. complanatum*, humans can be incidentally infected by eating raw or undercooked freshwater fish infected by *C. complanatum* metacercariae (Soylu 2013), causing parasitic pharyngitis and laryngitis (Gaglio et al. 2016). Human infections are rather rare and have occurred mainly in Asian countries (e.g. Korea) with a tradition of eating raw fish (Kim et al. 2019).

*Eustrongylides* sp. may also pose a public health risk to consumers of raw or undercooked fish, such as perch (Branciari et al. 2016). Human infections have been recorded mainly in Asia (Ljubojevic et al. 2015) or Africa (Eberhard and Ruiz-Tiben 2014). Although humans are not frequent hosts for species of *Eustrongylides*, it is known that this fish-borne zoonosis can cause gastritis and intestinal perforation in occasionally infected human. According to the recommendations of the European Commission, food producers should visually examine fish products before their release to the market (Branciari et al. 2016). Since larvae of species of *Eustrongylides* are typically large and are conspicuously red, they are easily differentiated from the fish tissue, even by visual inspection.

A potential risk of transmission of *C. complanatum* and *Eustrongylides* sp. from perch to humans in Europe is very limited, although it can not be absolutely excluded. A good example is diphyllobothriosis, fish-borne zoonosis, which re-emergence in the subalpine region was due to increased popularity of raw perch dishes (Wicht et al. 2009).

The Danube and its adjacent floodplain forests are characterized by rich aquatic and terrestrial faunas. However, anthropogenic activities, such as hydropower dams (Schiemer et al. 2004) may influence diversity and number of aquatic species (Guti 1992). The data on fish parasites from the Danube are, in general, scarce. Since some information are rather old and require updates, up-to-date surveys are necessary for accurate knowledge on fish parasites from this dynamically changing aquatic environment.
